# The effect of dopamine replacement therapy on cortical structure in Parkinson's disease

**DOI:** 10.1111/cns.14540

**Published:** 2023-11-23

**Authors:** Chenqing Wu, Haoting Wu, Cheng Zhou, Tao Guo, Xiaojun Guan, Zhengye Cao, Jingjing Wu, Xiaocao Liu, Jingwen Chen, Jiaqi Wen, Jianmei Qin, Sijia Tan, Xiaojie Duanmu, Luyan Gu, Zhe Song, Baorong Zhang, Peiyu Huang, Xiaojun Xu, Minming Zhang

**Affiliations:** ^1^ Department of Radiology, The Second Affiliated Hospital Zhejiang University School of Medicine Hangzhou China; ^2^ Department of Neurology, The Second Affiliated Hospital Zhejiang University School of Medicine Hangzhou China

**Keywords:** cortical structure, dopamine replacement therapy, MRI, parkinson's disease

## Abstract

**Aims:**

To explore the cortical structural reorganization in Parkinson's disease (PD) patients under chronic dopamine replacement therapy (DRT) in cross‐sectional and longitudinal data and determine whether these changes were associated with clinical alterations.

**Methods:**

A total of 61 DRT‐treated, 60 untreated PD patients, and 61 normal controls (NC) were retrospectively included. Structural MRI scans and neuropsychological tests were conducted. Cortical thickness and volume were extracted based on FreeSurfer and were analyzed using general linear model to find statistically significant differences among three groups. Correlation analyses were performed among significant cortical areas, medication treatment (duration and dosage), and neuropsychological tests. Longitudinal cortical structural changes of patients who initiated DRT were analyzed using linear mixed‐effect model.

**Results:**

Significant cortical atrophy was primarily observed in the prefrontal cortex in treated patients, including the cortical thickness of right pars opercularis and the volume of bilateral superior frontal cortex (SFC), left rostral anterior cingulate cortex (rACC), right lateral orbital frontal cortex, right pars orbitalis, and right rostral middle frontal cortex. A negative correlation was detected between the left SFC volume and levodopa equivalent dose (LED) (*r* = −0.316, *p* = 0.016), as well as the left rACC volume and medication duration (*r* = −0.329, *p* = 0.013). In the patient group, the left SFC volume was positively associated with digit span forward score (*r* = 0.335, *p* = 0.017). The left SFC volume reduction was longitudinally correlated with increased LED (standardized coefficient = −0.077, *p* = 0.001).

**Conclusion:**

This finding provided insights into the influence of DRT on cortical structure and highlighted the importance of drug dose titration in DRT.

## Graphical abstract

Significant cortical atrophy was primarily observed in the prefrontal cortex in DRT‐treated patients, including bilateral superior frontal cortex (SFC) and left rostral anterior cingulate cortex (rACC). the left SFC volume was negatively correlated with levodopa equivalency dose (LED), as well as the left rACC volume and medication duration.

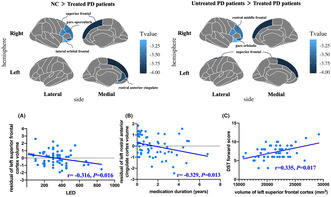

## 1 INTRODUCTION

Parkinson's disease (PD) is a heterogeneous neurological disorder that causes both motor and nonmotor symptoms.^1^, ^2^ The primary pathology involves dopaminergic neuron degeneration in the nigrostriatal pathway.^1^ Dopaminergic medication compensates for this deficiency, and dopamine replacement therapy (DRT) represents the standard treatment for over 50 years.^3^, ^4^ However, DRT cannot slow down the disease progression, and several patients would suffer from DRT‐related clinical symptoms, like motor complications, behavioral problems, and autonomic, emotional, and cognitive issues.^5^, ^6^, ^7^, ^8^ Evidence from fundamental research indicated increased immunoreactivity in the dopaminergic neural circuit with dendritic atrophy and increased receptor expression after chronic DRT exposure,^9^, ^10^, ^11^ suggesting that exogenous dopamine would influence brain performance in deep. Therefore, intrinsic brain organization may be reshaped in clinical PD patients under chronic DRT, which is largely unknown.

Magnetic resonance imaging (MRI) is one of the most popular techniques to explore brain pathophysiological changes in vivo. Functionally, chronic DRT up‐regulated functional connectivity within nigro‐striato‐cortical circuit,^12^ normalized regional activity in cerebellum and motor cortex,^13^ and reorganized executive and sensorimotor networks.^14^ Thus, with chronic exogenous dopamine exposure, the BOLD signal coupling detected by functional MRI is restructured to maintain “normal neural support”. It is worth noting that brain function is largely shaped by cortical structures, for example, neural plasticity, dendritic, and synaptic reorganization, which jointly consist of human brain structural underpinning.^15^, ^16^ However, whether chronic DRT would intrinsically influence brain structure remains unknown. Up‐to‐date, high‐resolution structural MRI is the best marker to objectively quantify whole brain gray matter as a metric of neuron and related components,^17^, ^18^ which has been well established in detecting PD degeneration,^19^ even in revealing chronic medication effects in schizophrenia and major depression disorders.^20^, ^21^ Taken together, the understanding of the way that chronic DRT reorganized brain structure would contribute to better clarifying PD pathophysiology and then explaining the alterations of patient behavior.

In this study, we comprehensively explored the cortical structural reorganization in PD patients under chronic DRT, longitudinally observed their dynamic alterations, and analyzed the potential associations with clinical symptoms. We hypothesized that chronic DRT would contribute to regional cortical atrophy, which was associated with dopaminergic medication duration and dosage.

## 2 MATERIALS AND METHODS

### 2.1 Participants

This research was approved by the hospital Ethics Committee and informed consent forms were obtained from all participants in accordance with the Declaration of Helsinki.

**FIGURE 1:**
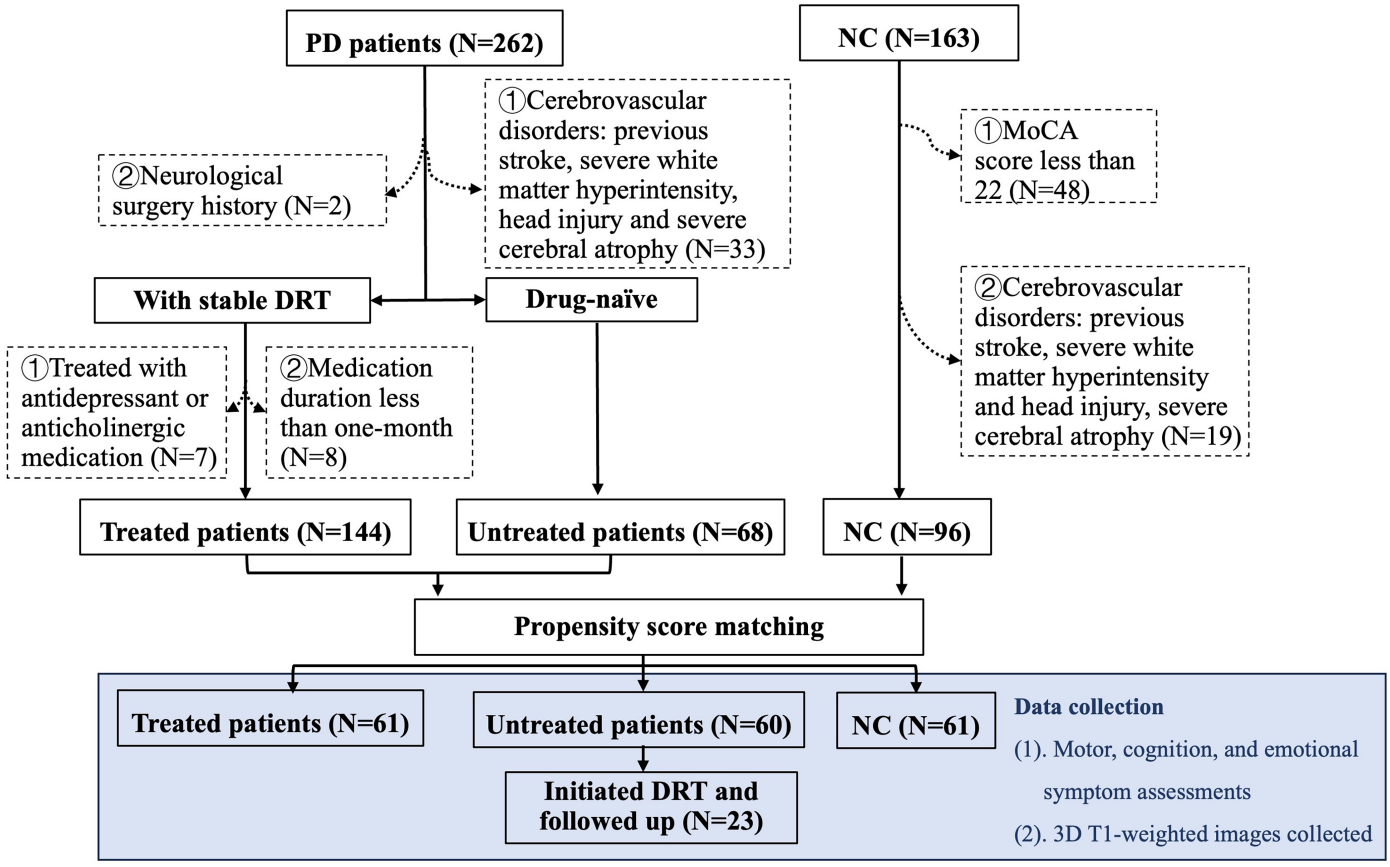
Inclusion and exclusion criteria and data collection of NC, treated and untreated patients. DRT, dopamine replacement therapy; MoCA, Montreal Cognitive Assessment; NC, normal controls; PD, Parkinson's disease.

A total of 262 PD patients were initially diagnosed by an expert neurologist according to the UK Parkinson's Disease Society Brain Bank diagnostic criteria and Movement Disorder Society clinical diagnostic criteria.^22^, ^23^ Patients were excluded due to ① cerebrovascular disorders, including stroke, severe white matter hyperintensity, head injury, and severe cerebral atrophy (*N* = 33);② neurological surgical history (*N* = 2);③ medication duration less than 1 month (*N* = 8) according to previous studies^24^, ^25^;④ treatment with antidepressant or anticholinergic medication (*N* = 7) as they could potentially influence cortical structure^21^, ^26^ (Figure 1). Treated patients were under stable chronic DRT for at least 1 month,^24^, ^25^ while untreated patients have not received any medication treatments for PD since their diagnosis. Meanwhile, 115 NCs were recruited from the community with a MoCA score of no less than 22.^27^ NC with cerebrovascular disorders was also excluded (*N* = 19) (Figure 1). Due to data imbalance among NC, treated, and untread groups (*N* = 96, 144, and 68, respectively), propensity score matching was performed to minimize the discrepancies of age, gender, disease duration, and H‐Y.^28^ This method ensures an even distribution of confounders among groups and increases group comparability.^28^ Finally, 61 DRT‐treated, 60 untreated patients and 61 NC were included in the final analysis.

Clinical assessments and MRI scans were conducted during OFF states (at least 12 h after withdrawing PD medication). Unified Parkinson's Disease Rating Scale (UPDRS) and Hoehn–Yahr stage (H‐Y) were performed to evaluate the motor symptoms. Cognitive assessments included Montreal Cognitive Assessment (MoCA) and Mini‐mental State Examination (MMSE) for global cognition, Boston naming test (BNT) for language, Digit Span Test (DST) and Symbol Digit Modality Test (SDMT) for attention and working memory, and Semantic Fluency Test (SFT) for executive function according to previous studies.^29^, ^30^ The severity of depression and anxiety symptoms was measured using Geriatric Depression Scale and Hamilton Anxiety Scale. The duration of dopaminergic medication and the kinds of medication were collected. The levodopa equivalent dose (LED) was calculated according to the previous statement.^31^ Medication therapy was adjusted to a stable level for optimal motor control and minimal side effects.

Twenty‐three patients (15 male, 58.87 ± 7.27 years old) of the 60 untreated patients initiated DRT after the baseline visit and were followed up (medium follow‐up time = 2.28 years). None of them received anticholinergic or antidepressant medications during the entire observational period. Clinical assessments and MRI scans at baseline and the last visit were used for analyses.

### 2.2 MRI acquisition

All imaging data was acquired on a 3‐Tesla MRI scanning system (Discovery MR750, GE Healthcare). Each participant's head was stabilized with foam pads. Earplugs were provided to diminish audible noise during scanning. Three‐dimensional T1‐weighted images were acquired with a fast spoiled gradient‐recalled sequence using following parameters: echo time = 3.036 ms; repetition time = 7.336 ms; inversion time = 450 ms; flip angle = 11 degrees; field of view = 260 × 260 mm^2^; matrix = 256 × 256; slice thickness = 1.2 mm; slice ga*p* = 0 mm; number of slices = 196.

### 2.3 MRI preprocessing and analyses

All parcellations were performed with fully automated segmentation software FreeSurfer (version 6.0.0, http://surfer.nmr.mgh.harvard.edu/) using “recon‐al” pipeline which included an automated procedure of motion correction, skull stripping, spatial normalization, registration, cortical parcellation, and volumetric segmentation. To ensure quality control, we visually inspected the segmentations of 68 cortical regions based on the Desikan‐Killiany atlas.^32^ Total intracranial volume (TIV) and bilateral cortical thickness and volume were extracted from the ROIs defined in the Desikan‐Killiany atlas.

The structural MR images of follow‐up patients were further processed according to FreeSurfer longitudinal processing framework.^33^ First, images were independently processed using “recon‐all” pipeline as described above, and then a within‐subject template was created. Several processing steps including skull stripping, registration, and parcellations were modified based on common information from this template, which allowed equal and unbiased treatments of all input images instead of using a particular time‐point as reference. After processing, the longitudinal data was visually checked for corresponding alignment between different time points.

### 2.4 Statistical analyses

Statistical analyses were performed using IBM SPSS Statistics software (version 26.0). The one‐sample Kolmogorov–Smirnov test was used to verify whether a continuous variable comes from a normal distribution. Continuous variables with normal distribution were presented as mean and standard deviation (SD) and compared using the independent sample *t* test or one‐way analysis of variance (ANOVA). Post‐hoc least significant difference (LSD) tests were performed after ANOVA. Continuous variables with nonparametric distribution were reported as median and interquartile range and compared using the Wilcoxon rank‐sum test or Kruskal‐Wallis test. Chi‐squared test was used for categorical variable analyses. A two‐tailed *p*‐value of <0.05 was considered as statistically significant.

Cortical volume and thickness differences among treated, untreated patients, and NC were analyzed using the general linear model (GLM) with age and gender as covariates. TIV was added as a covariate for cortical volume analysis. False discovery rate (FDR) was used for multiple comparison corrections and the *Q*‐value was presented.^34^

Partial correlation was performed between cortical regions showing significant inter‐group differences and medication treatment (duration and LED), controlling for age, gender, and disease duration. Besides, the association between statistically significant cortical regions and clinical symptoms (motor, cognition, and emotion) was analyzed, controlling for age, gender, disease duration, and year of education. TIV was controlled in cortical volume analysis.

Next, to pinpoint the specific effects of DRT on cortical structure, we explored whether medication duration and dosage (LED) differently affected the significant cortical regions observed in the GLM analysis described above. The median split method was used to dichotomize treated patients into high‐LED (long‐duration) and low‐LED (short‐duration) groups. Analyses of covariance (ANCOVAs) and post hoc tests (LSD adjustment) were used to find cortical structural differences with the LED group (high‐LED and low‐LED), medication duration group (long‐duration and short‐duration) and their interaction (LED×medication‐duration) as fixed factors, controlling for age and gender. TIV was controlled in cortical volume analysis.

Linear mixed‐effect (LME) model was used to analyze the relationship between medication treatment (duration and dosage) and longitudinal changes of the significant cortical regions found in the cross‐sectional study. Fixed effects included medication duration and LED as the main effects, and age, gender, and disease duration as covariates (TIV was added for volume analysis). Intercepts for participants and slopes for disease duration time were entered as random effects. As exploratory analyses, LME models were also employed to analyze changes induced by DRT treatment in all 136 cortical structures with the same main effects and covariates as mentioned above.

Additional analysis was carried out to compare the cross‐sectional T‐values between treated and untreated patients to the longitudinal T‐values between untreated PD patients at baseline and after DRT treatment. Cross‐sectional and longitudinal T‐values in 136 cortical structures were recorded and Pearson's correlation was used to compare the relationship between these two T‐values.

## 3 RESULTS

### 3.1 Participants' characteristics

The demographic, clinical characteristics, and TIV are summarized in Table 1. The detailed information about dopaminergic medication in treated groups was summarized in Table S1. No significant difference was found among NC, untreated, and treated patients in age, gender, and year of education. The UPDRS III score of the treated patients was significantly lower than that of untreated ones (*p* = 0.022). Compared to NC, patients showed significantly worse performance in multiple cognitive tests including MMSE, MoCA, SF, SDMT, and BNT (*p* < 0.01) and more severe anxiety symptoms (*p* < 0.001), while no significant difference was observed between treated and untreated patients. The treated patient showed reduced TIV than untreated patients (*p* = 0.008) and NC (*p* = 0.008).

**TABLE 1:** Demographic and clinical data of NC, untreated and treated PD groups.

	untreated PD (*N* = 60)	treated PD (*N* = 61)	NC (*N* = 61)	*p*	Post‐hoc analyses *p* value (LSD)		
					Untreated PD vs treated PD	Untreated PD vs NC	Treated PD vs NC
Gender (M/F)	33/27	29/32	33/28	0.668			
Age (years)	58.07 ± 10.16	58.46 ± 8.93	58.18 ± 6.38	0.968			
Education (years)	9.00 (5.00–12.00)	9.00 (5.50–12.00)	9.00 (7.50–12.00)	0.055			
Cognitive tests							
MMSE	28.00 (25.00–29.00)	28.00 (26.00–29.00)	29.00 (28.00–30.00)	<0.001*	1.000	<0.001*	<0.001*
MoCA^a^	23.00 (18.00‐27.00)	23.00 (19.00–26.00)	26.00 (24.00–28.00)	<0.001*	1.000	0.001*	<0.001*
DST forward^b^	7.00 (6.00–9.00)	7.00 (6.00–8.00)	8.00 (6.00–9.00)	0.259			
DST backward^b^	4.00 (3.00–5.00)	3.50 (2.00–5.00)	4.50 (4.00–5.00)	0.149			
DST total^b^	11.00 (9.00–14.00)	10.50 (8.00–13.00)	13.00 (10.00–14.00)	0.162			
SF^c^	14.00 (11.50–19.50)	14.00 (12.00–89.00)	18.00 (14.00–21.00)	0.004*	1.000	0.021*	0.010*
SDMT^b^	33.00 (23.00‐48.00)	31.50 (25.25–49.00)	43.00 (34.00–57.00)	0.007*	1.000	0.016*	0.045*
BNT^b^	22.00 (19.50‐22.50)	22.00 (21.00–23.75)	26.00 (23.0–28.00)	<0.001*	0.345	<0.001*	0.001*
GDS	2.00 (1.00–4.00)	2.00 (1.00–4.00)	1.00 (0.00–3.00)	0.243			
HAMA	4.00 (2.00–8.75)	4.00 (2.00–6.00)	2.00 (0.00–4.00)	<0.001*	1.000	<0.001*	<0.001*
Disease duration (years)	2.18 (1.02–3.40)	2.39 (1.26–3.56)	/	0.881			
H‐Y	2.00 (2.00–2.50)	2.50 (1.50–2.50)	/	0.594			
UPDRS III	19.00 (14.00–31.00)	17.00 (11.00–23.00)	/	0.022*			
LED	/	320.53 ± 179.37	/	/			
Medication duration (years)	/	1.13(0.30–2.66)	/	/			
TIV	1535353.02 ± 157819.37	1465288.65 ± 143536.83	1534713.004 ± 146636.97	0.010*	0.008*	0.980	0.008*

Abbreviations: BNT, Boston naming test; DST, digit span test; GDS, Geriatric Depression Scale; HAMA, Hamilton Anxiety Scale; H‐Y, Hoehn–Yahr stage; LED, levodopa equivalent dose; MMSE, Mini‐mental State Examination; MoCA, Montreal Cognitive Assessment; SDMT, Symbol Digit Modality; SF, semantic fluency test; TIV, total intracranial volume; UPDRS, Unified Parkinson's Disease Rating Scale.

^a^ Results of 44 untreated PD, 60 treated PD and 61 NC.

^b^ Results of 29 untreated PD, 26 treated PD and 45 NC.

^c^ Results of 33 untreated PD, 30 treated PD and 45 NC.

* Statistically significant with *p*<0.05.

The median LED and medication duration of treated patients were 350 and 1.13 years respectively, based on which they were subdivided into four groups. No significant difference was found among four subgroups in demographic and clinical data except for age (*p* = 0.040) and disease duration (*p* < 0.001) (Table 2), therefore combined with age, gender, and TIV, disease duration was added as a covariate in ANCOVAs.

**TABLE 2:** Demographic and clinical data of the four median‐split treated groups.

	Short duration with low LED (*N* = 20)	Long duration with low LED (*N* = 12)	Short duration with high LED (*N* = 10)	Long duration with high LED (*N* = 19)	*p* value
Gender (M/F)	12/8	3/9	3/7	11/8	0.116
Age (years)	55.00 ± 7.35	56.28 ± 10.64	63.27 ± 9.19	60.94 ± 7.85	0.040* ^,^ ^#^
Education (years)	9.00 (5.25–12.00)	9.00 (6.00–12.00)	9.50 (5.25–12.00)	6.00 (4.00–9.00)	0.541
MMSE	28.00 (25.50–29.75)	28.50 (27.00–30.00)	28.00 (26.00–28.50)	27.00 (25.00–28.00)	0.483
MoCA	24.00 (22.00–27.00)	24.50 (22.00–26.75)	21.00 (18.75–26.00)	21.00 (17.00–25.00)	0.131
GDS	2.00 (0.50–4.00)	3.00 (0.75–7.75)	3.00 (1.25–4.75)	1.50 (0.25–4.00)	0.695
HAMA	4.00 (2.00–6.00)	4.00 (3.25–11.00)	4.50 (2.25–9.00)	4.00 (2.00–10.00)	0.876
Disease duration (years)	1.06 (0.57–1.84)	3.02 (2.30–3.70)	1.74 (1.29–3.65)	3.56 (2.72–4.81)	<0.001* ^,^ ^#^
H‐Y	2.00 (1.63–2.50)	2.25 (1.50–2.50)	2.50 (1.50–2.50)	2.50 (2.00–2.50)	0.847
UPDRS III	14.60 ± 6.61	17.67 ± 5.85	19.00 ± 8.50	21.84 ± 13.43	0.133
LED	186.90 ± 96.44	241.75 ± 60.30	410.00 ± 54.26	472.37 ± 146.22	<0.001^*^
Medication duration (years)	0.22 (0.10–0.46)	2.15 (1.42–3.16)	0.38 (0.19–0.83)	2.89 (1.98–4.43)	<0.001^*^

*Note*: (1) age: ①short duration with low LED vs short duration with high LED *p* = 0.015; ②short duration with low LED vs long duration with high LED *p* = 0.034; other comparisons were not statistically significant.

(2) disease duration: ①short duration with low LED vs long duration with low LED *p* = 0.001; ②short duration with low LED vs long duration with high LED *p*<0.001; other comparisons were not statistically significant.

Abbreviations: DST, digit span test; H‐Y, Hoehn–Yahr stage; LED, levodopa equivalent dose; MMSE, Mini‐mental State Examination; MoCA, Montreal Cognitive Assessment; SF, semantic fluency test; TIV, total intracranial volume; UPDRS, Unified Parkinson's Disease Rating Scale.

* Statistically significant with *p*<0.05.

^#^ Post‐hoc analyses (LSD).

### 3.2 Inter‐group differences in cortical structures

**FIGURE 2:**
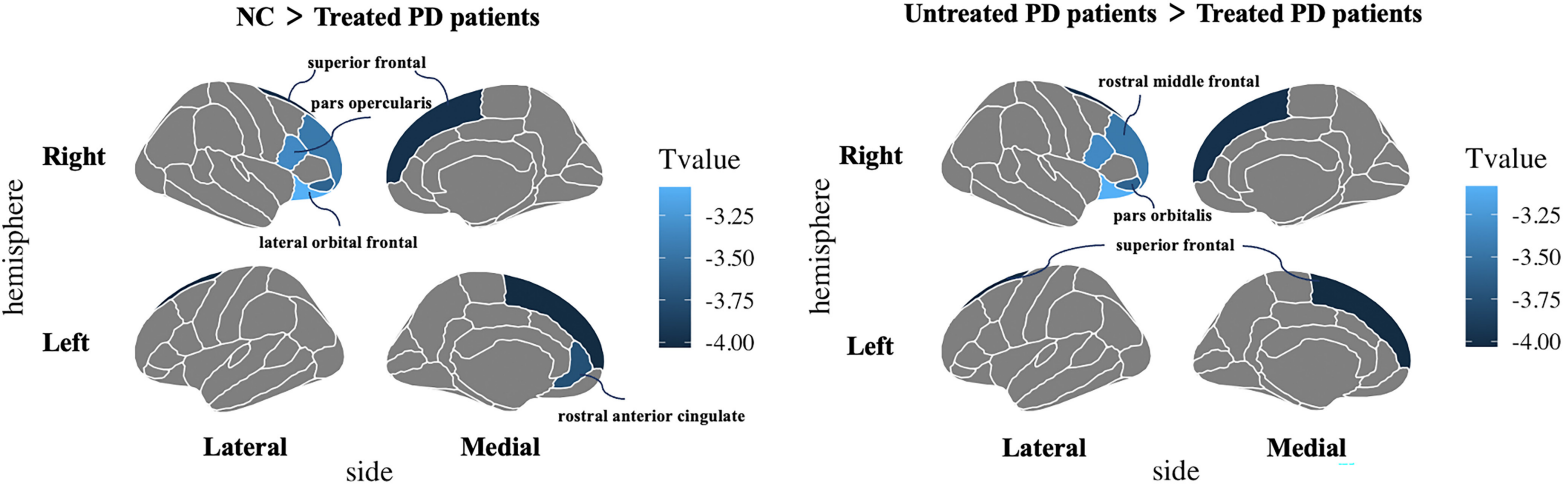
Inter‐group differences in cortical structures. Differences in cortical thickness or volume among NC, treated, and untreated patients. Lower volume or cortical thickness is indicated by blue color. Significant cortical structures include the cortical thickness of the right pars opercularis, and the volume of the bilateral superior frontal cortex, left rostral anterior cingulate cortex, right lateral orbital frontal cortex, right pars orbitalis, and right rostral middle frontal cortex. NC, normal controls; PD, Parkinson's disease.

The brain regions demonstrated significant differences among NC, treated and untreated patients were mainly located in the prefrontal region, including bilateral superior frontal cortex (SFC), left rostral anterior cingulate cortex (rACC), right rostral middle frontal cortex, right pars opercularis, right pars orbitalis and right lateral orbital frontal cortex (*Q value* <0.050) (Figure 2 and Table S2). The post‐hoc analyses showed that the treated patients demonstrated a significant cortical volume reduction than untreated patients and NC in bilateral SFC, left rACC, right rostral middle frontal cortex, right pars orbitalis, and right lateral orbital frontal cortex (*p* < 0.010, LSD adjusted). The treated patients had lower cortical thickness in the right pars opercularis cortex compared to NC and untreated patients (*p* < 0.050, LSD adjusted). No significant difference in cortical volume and thickness was found between untreated patients and NC after multiple comparison corrections.

### 3.3 Correlations among cortical structural changes, medication duration, LED, and neuropsychological tests

**FIGURE 3:**
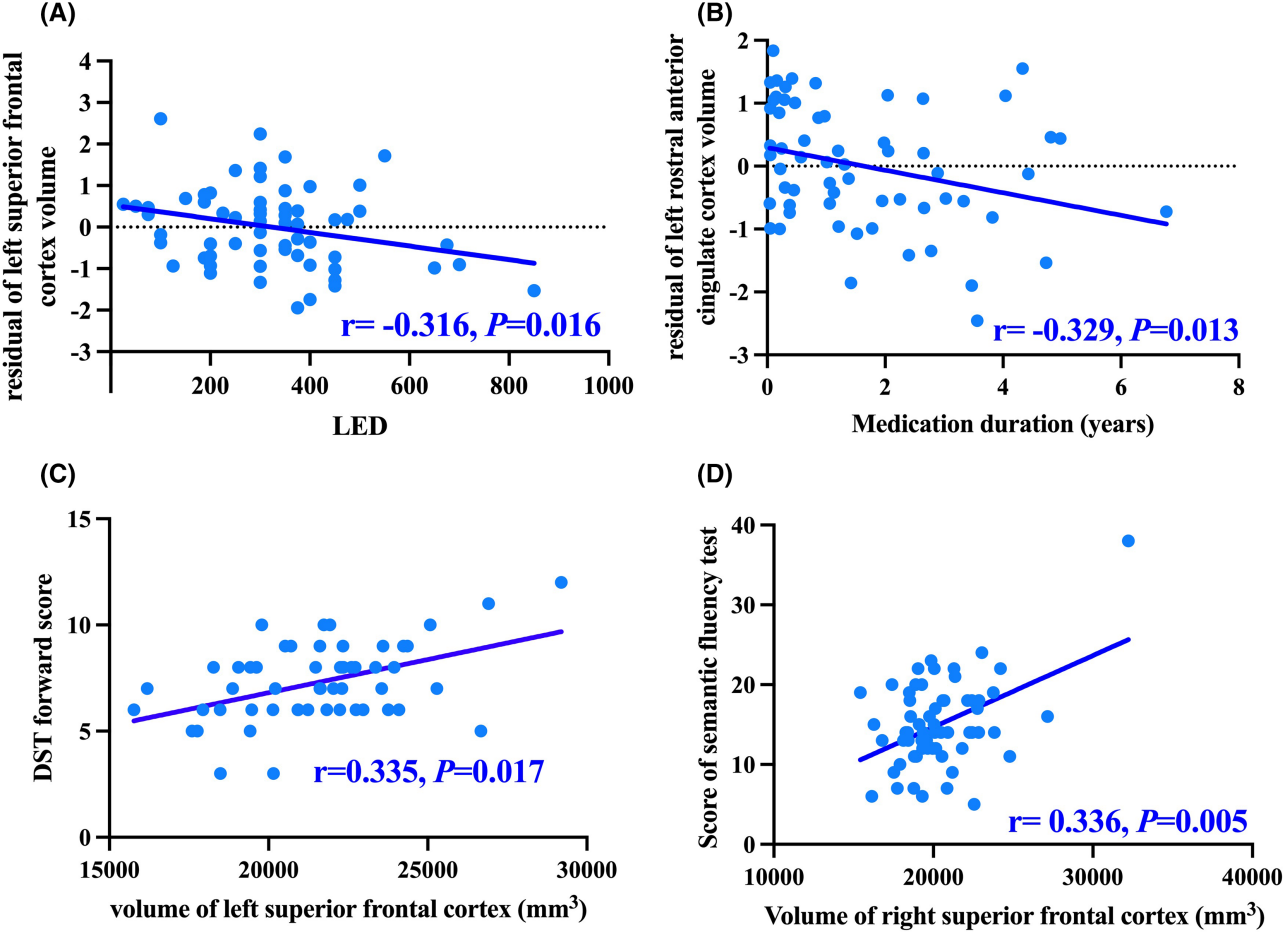
Correlations among cortical structural changes, medication duration, LED, and neuropsychological tests. (A) The left superior frontal cortex volume was negatively correlated with LED (*r* = −0.316, *p* = 0.016, *Q* = 0.072). (B) The left rostral anterior cingulate cortex volume was negatively correlated with medication duration (*r* = −0.329, *p* = 0.013, *Q* = 0.088). (C) The left SFC volume was positively associated with the score of the DST forward test in all PD patients (*r* = 0.335, *p* = 0.017, *Q* = 0.122) (D) A significant correlation between the right superior frontal cortex volume and the score of the semantic fluency test was observed (*r* = 0.336, *p* = 0.005, *Q* = 0.034). DST, digit span test; LED, levodopa equivalent dose.

The left SFC volume was negatively correlated with LED (*r* = −0.316, *p* = 0.016, *Q* = 0.072), and the left rACC volume was negatively correlated with medication duration (*r* = −0.329, *p* = 0.013, *Q* = 0.088) (Figure 3A,B, Table S3). However, these results failed to pass FDR correction probably due to the relatively small sample size in our study. Additionally, a significant correlation between the right SFC volume and the score of the semantic fluency test was observed (*r* = 0.336, *p* = 0.005, *Q* = 0.034). The left SFC volume was positively associated with the score of the DST forward test in all PD patients (*r* = 0.335, *p* = 0.017, *Q* = 0.122) (Figure 3C,D), though failed to pass the FDR correction.

### 3.4 Effects of LED and medication duration on significant cortical structures

**FIGURE 4:**
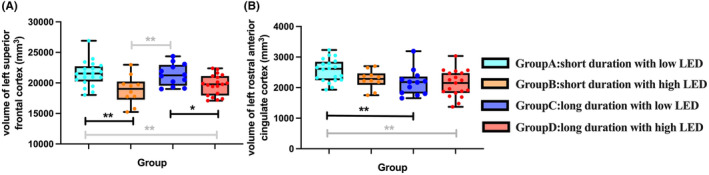
Effects of LED and medication duration on significant cortical regions. Group differences within the four median‐split treated groups. (A) Patients in Group B showed a significant reduction in the left superior frontal cortex volume than those in Group A (*p* = 0.001). Such LED‐related effect diminished between two long medication duration groups (Group C and Group D, *p* = 0.022). (B) Of the two low‐LED groups, patients in Group C had significantly reduced left rostral anterior cingulate cortex volume than patients in Group A (*p* = 0.009). *p<0.05, **p<0.01. LED, levodopa equivalent dose.

After dichotomizing treated patients based on median LED/medication duration, significant main effects of LED group (high and low LED) on left SFC volume (F (1, 51)=4.402, *p* = 0.041) and medication duration (long and short duration) on left rACC volume (F (1, 51)=9.917, *p* = 0.003) were observed. However, no significant *LED×medication duration* interaction was found on left SFC (F (3, 51)=0.117, *p* = 0.734) or rACC volume (F (3, 51)=0.210, *p* = 0.648). Post‐hoc analyses revealed that within two short medication duration groups, patients with high LED showed a significant reduction in the left SFC volume than those with low LED (*p* = 0.001, LSD adjusted) (Figure 4A, Table S4). Such LED‐related effects diminished in long medication duration groups (*p* = 0.022, LSD adjusted). Of the low‐LED groups, patients with long medication duration had significantly reduced left rACC volume than patients with short duration (*p* = 0.009, LSD adjusted) (Figure 4B). In patients with high LED, such difference in left rACC volume was not significant between long and short medication duration groups (*p* = 0.353, LSD adjusted).

### 3.5 Longitudinal analysis of cortical structural changes

The medium follow‐up time was 2.28 years, the medium LED was 400, and the medium medication duration was 2.25 years (Table S5). The longitudinal reduction of the left SFC volume was significantly associated with increased LED (standardized coefficient = −0.077, 95% confidence interval: −0.122 ~ −0.032, *p* = 0.001), which was consistent with former cross‐sectional observation (Table 3). No significant correlation was observed between the left rACC volume and medication duration in the longitudinal analysis. The significant results of longitudinal cortical changes related to DRT in all 136 cortical structures are summarized in Tables S6–S7 and Figure S1. There was a significant correlation between cross‐sectional *T* values and longitudinal *T* values in 136 cortical structures (*r* = 0.228, *p* = 0.007) (Figure S2).

**TABLE 3:** Results of the linear mixed‐effect model in the longitudinal data.

Fixed effect	Standardized coefficient	95% Confidence interval		*T* value	*p* value
		lower	upper		
(Intercept)	0.253	−0.262	0.768	0.993	0.327
Gender (male)	−0.390	−1.087	0.308	−1.130	0.265
Age	−0.087	−0.355	0.181	−0.658	0.514
Disease duration	−0.236	−0.505	0.032	−1.780	0.083
LED	−0.077	−0.122	−0.032	−3.441	0.001*
Drug duration	0.080	−0.105	0.266	0.878	0.386
TIV	0.786	0.454	1.119	4.780	<0.001*

Abbreviations: LED, levodopa equivalent dose; TIV, total intracranial volume.

* Statistically significant with *p* < 0.05.

## 4 DISCUSSION

This study investigated the cortical reorganization in PD patients under chronic DRT cross‐sectionally and longitudinally. Comparing to untreated patients, patients under chronic DRT showed significant prefrontal cortical atrophy including SFC and rACC. In addition, the left SFC and rACC atrophy were respectively correlated with increased LED and medication duration. The left SFC volume was positively associated with DST forward score in all patients.

In treated patients, significant atrophy in the prefrontal cortex (PFC), including SFC and rACC, was observed. The PFC is critical for multiple higher‐order cognitive function including planning, attention and working memory; and the dopaminergic metabolism in this region was closely associated with cognitive function in non‐demented PD.^35^, ^36^, ^37^ Importantly, the PFC atrophy was correlated with increased dosage and longer duration of DRT, which was also observed in longitudinal analyses. Multiple fundamental and clinical studies supported the chronic dopamine administration had a detrimental effect on PFC. Chronic DRT exposure increased immunoreactivity and astrocytosis in PFC with neuron dendritic atrophy and caused working memory impairment in Parkinsonian rats, reflecting maladaptive neural response to chronic dopamine exposure.^11^, ^38^ In vivo, young adults with higher cortical dopamine levels showed lower PFC volume and performed worse in working memory tasks after levodopa challenge.^39^, ^40^ The “Dopamine overdose hypothesis” provided a plausible interpretation, which assumed underlying adverse effects of excessive dopamine on PFC and working memory function.^37^, ^41^ The overdose effects are assumed to be prominent in the early stage and higher LED could potentially exert a negative influence on prefrontal function, as characterized by the right half of the U‐inverted curve.^42^ Because the dopaminergic neurons in the ventral tegmental area were relatively less affected than those in the substantia nigra at early‐stage PD, and dopaminergic medication may lead to relative “overdose” in the less affected mesocortical dopaminergic pathway projecting to the frontal cortex and induce negative impacts.^36^, ^41^ The negative correlations between LED and frontal cortex volume in our observation are consistent with this hypothesis. PFC activation followed an “inverted‐U” dose–response, that is, either too little or too much dopamine stimulation would impair PFC function.^42^, ^43^ In summary, our finding reflected the detrimental influence of chronic dopamine exposure on PFC in PD and provided solid evidence for “Dopamine overdose hypothesis” in vivo. However, the negative correlation between rACC volume and medication duration was only observed in the cross‐sectional but not longitudinal data. The possible reason for such inconsistency might be higher LED of follow‐up patients (medium LED = 400) than those in the cross‐sectional cohort (medium LED = 350) because in the cross‐sectional data, the significant difference in left rACC volume between the long and short medication duration groups was only observed in patients with low LED (LED less than 350) but not those with high LED.

No significant cortical atrophy was found in untreated patients compared to NC in this study. Frontal and occipito‐parietal cortical atrophy in PD patients was reported in previous studies, but patients enrolled in these studies had much longer disease duration (6–8 years) than those in our study (median duration 2.30 years).^19^, ^44^ Consistent with current findings, when recruiting untreated patients with shorter disease duration (1.32 years on average), no cortical volume difference was observed between patients and NC.^45^ Therefore, possibly when drug‐naïve PD patients are at their early stages, structural atrophy may be not the main pathological change, or the technique is not sensitive enough to detect the alteration.

Additionally, the volume of SFC was associated with the DST forward test score which reflected working memory function. The SFC was located at the superior part of PFC.^46^ As well documented, PFC atrophy was significantly associated with impaired working memory.^47^, ^48^, ^49^ Patient(s) with PFC lesions restricted to the left SFC would exhibit working memory deficits, while after the direct cortical stimulation of the left SFG, epilepsy patients showed an enhancement in working memory performance.^50^, ^51^ However, no significant difference in cognitive performance between treated and untreated PD patients was observed in the presented study. Chronic DRT was not enough to sustainably compensate for cognitive impairment in PD and the cognitive performance gradually declined after the first 1.5 years medication treatment.^52^ Additionally, brain network reorganization in frontal region was reported in patients under chronic DRT, reflecting possible functional compensation for cognitive performance.^14^ Taken together, PFC atrophy was correlated with worse working memory function in treated patients, but this preliminary finding should be longitudinally investigated to confirm its association with cognition prognosis.

There were several limitations in this study. First, as most untreated patients initiated DRT soon after clinical diagnosis, longitudinal data of untreated patients was unavailable and it is hard to compare the difference in longitudinal cortical structural changes between treated and untreated patients. Second, only structural MRI was analyzed in this research, it is crucial to integrate other neuroimaging methods and biological markers for a comprehensive understanding of chronic DRT influence. Third, although negative correlations between LED and cortical structures were observed in this study, the potential presence of other statistical relationships, such as U or U‐inverted relationships, should be taken into consideration in the future to comprehensively illustrate the influence of dopaminergic medication on the cortex. Fourth, other cognitive assessments such as the Clock Drawing Test and cube copy for visuospatial function and Trail Making Test for executive function were also performed, but these tests could be influenced by the severity of motor symptoms. Some patients with severe bradykinesia or tremors failed to complete these tests in this study. In the future, a more comprehensive assessment of cognitive domains that are primarily affected by PD should be analyzed. Last, parts of treated patients included were prescribed different kinds of dopaminergic medication, including dopamine agonists and monoamine oxidase type‐B inhibitors. Although LED and medication duration were recorded to measure the overall dopaminergic medication effects and propensity score matching was used to minimize the discrepancies of confounding factors, randomized controlled trials should be further carried out to explore the relationship between dopaminergic medication and cognition.

In conclusion, significant PFC atrophy was found in patients under chronic DRT, and higher LED and longer medication duration would contribute to this effect. Besides, the SFC volume was positively related to working memory performance. These findings provided novel insights into the influence of chronic dopaminergic medication on cortical structure and its possible detrimental effect on working memory. These results also highlighted the importance of drug dose titration in DRT to maintain a balance between symptom remission and possible deleterious effects.

## CONFLICT OF INTEREST STATEMENT

The authors have declared no conflict of interest.

## Supporting information


Data S1.

## Data Availability

The materials used and/or analyzed during the current study are available from the corresponding author on reasonable request.
