# Supplementary material for: The effect of dopamine replacement therapy on cortical structure in Parkinson's disease
Source: CNS Neurosci Ther. 2023 Nov 23;30(4):e14540. doi: 10.1111/cns.14540 (PMC11017430; doi:10.1111/cns.14540)
Supplement: Supplementary file 1 — Data S1. [file CNS-30-e14540-s001.docx]

**Supplementary Table 1. Detailed information about dopaminergic medication in treated groups**

| **Medication** | **Number of patients (ALL=61)** | **LED**  **(Mean±SD)** | **Medication duration**  **(Median and interquartile range, years)** |
| --- | --- | --- | --- |
| **Levodopa** | 15 | 370±137.32 | 0.97 (0.21-2.52) |
| **DA** | 7 | 130.43±100.53 | 0.38 (0.25-0.71) |
| **MAO-B inhibitors** | 3 | 100±0 | 0.15 (0.10-0.18) |
| **Levodopa + DA** | 17 | 362.53±118.70 | 1.95 (0.63-2.89) |
| **Levodopa + MAO-B inhibitors** | 10 | 300.00±78.17 | 1.25 (0.36-1.49) |
| **DA + MAO-B inhibitors** | 2 | 156.50±44.55 | 1.94 (1.57-2.30) |
| **Levodopa + DA + MAO-B inhibitors** | 7 | 496.43±196.02 | 4.43 (2.71-4.89) |
| DA, dopamine agonist; LED, levodopa dose equivalent dose; MAO-B, Monoamine Oxidase Type-B; SD, standard deviation. | | | |

**Supplementary Table 2. Significant cortical thickness and volume differences among NC, treated, and untreated patients.**

| **Measurement** | **Hemisphere** | **Region** | ***F***  **value** | ***Q* value (FDR corrected)** | **Post-hoc *P* value (LSD)** | | |
| --- | --- | --- | --- | --- | --- | --- | --- |
|  |  |  |  |  | **untreated PD＞treated PD** | **NC＞**  **untreated PD** | **NC＞treated PD** |
| **Cortical thickness** | **R** | **Pars opercularis** | 11.884 | 0.023 | **0.022** | 0.334 | **0.001** |
| **Volume** | **L** | **Rostral anterior cingulate cortex** | 14.594 | 0.011 | 0.132 | 0.074 | **< 0.001** |
|  |  | **Superior frontal cortex** | 18.215 | 0.007 | **< 0.001** | 0.144 | **0.001** |
|  | **R** | **Lateral orbital frontal cortex** | 10.356 | 0.046 | **< 0.001** | 0.495 | **0.001** |
|  |  | **Pars orbitalis** | 14.051 | 0.014 | **0.001** | 0.332 | **0.001** |
|  |  | **Rostral middle frontal cortex** | 12.186 | 0.019 | **< 0.001** | 0.461 | **< 0.001** |
|  |  | **Superior frontal cortex** | 16.489 | 0.007 | **< 0.001** | 0.315 | **< 0.001** |

FDR, false discovery rate; L, left; LSD, least significant difference; NC, normal control; PD, Parkinson’s disease; R, right.

**Supplementary Table 3. Correlation analysis between cortical structures and clinical variables**

| **Clinical variables** | **Statistic values** | | **Volume** | | | | | | | | | | **Thickness** | |
| --- | --- | --- | --- | --- | --- | --- | --- | --- | --- | --- | --- | --- | --- | --- |
|  |  |  | **lh_rostralanteriorcingulate** | | | **lh_superiorfrontal** | **rh_lateralorbitofrontal** | | **rh_parsorbitalis** | **rh_rostralmiddlefrontal** | | **rh_superiorfrontal** | | **rh_parsopercularis** |
| **MMSE** | | **R value** | | -0.076 | -0.040 | | -0.067 | -0.128 | | | -0.101 | 0.049 | | -0.095 |
|  |  | **P value** | | 0.415 | 0.671 | | 0.472 | 0.172 | | | 0.281 | 0.598 | | 0.312 |
|  |  | **Q value** | | 0.661 | 0.671 | | 0.661 | 0.661 | | | 0.661 | 0.671 | | 0.661 |
| **MoCA** | | **R value** | | -0.138 | 0.013 | | -0.129 | -0.131 | | | -0.010 | -0.009 | | -0.052 |
|  |  | **P value** | | 0.173 | 0.895 | | 0.205 | 0.195 | | | 0.923 | 0.927 | | 0.608 |
|  |  | **Q value** | | 0.477 | 0.927 | | 0.477 | 0.477 | | | 0.927 | 0.927 | | 0.927 |
| **SF** | | **R value** | | -0.138 | 0.038 | | 0.046 | -0.041 | | | 0.068 | 0.365 | | 0.080 |
|  |  | **P value** | | 0.301 | 0.777 | | 0.730 | 0.761 | | | 0.611 | **0.005*** | | 0.549 |
|  |  | **Q value** | | 0.777 | 0.777 | | 0.777 | 0.777 | | | 0.777 | **0.034*** | | 0.777 |
| **DST forward** | | **R value** | | 0.014 | 0.335 | | 0.096 | 0.088 | | | 0.129 | 0.252 | | 0.092 |
|  |  | **P value** | | 0.923 | **0.017*** | | 0.505 | 0.543 | | | 0.371 | 0.077 | | 0.526 |
|  |  | **Q value** | | 0.923 | 0.122 | | 0.633 | 0.633 | | | 0.633 | 0.271 | | 0.633 |
| **DST backward** | | **R value** | | 0.069 | 0.090 | | -0.098 | -0.231 | | | -0.089 | 0.041 | | -0.210 |
|  |  | **P value** | | 0.636 | 0.533 | | 0.500 | 0.107 | | | 0.538 | 0.777 | | 0.142 |
|  |  | **Q value** | | 0.742 | 0.742 | | 0.742 | 0.497 | | | 0.742 | 0.777 | | 0.497 |
| **SDMT** | | **R value** | | -0.165 | -0.049 | | -0.098 | -0.041 | | | -0.123 | -0.212 | | -0.148 |
|  |  | **P value** | | 0.274 | 0.747 | | 0.515 | 0.786 | | | 0.416 | 0.158 | | 0.327 |
|  |  | **Q value** | | 0.721 | 0.786 | | 0.721 | 0.786 | | | 0.721 | 0.721 | | 0.721 |
| **LED** | | **R value** | | -0.186 | -0.316 | | -0.232 | 0.088 | | | 0.045 | -0.118 | | -0.306 |
|  |  | **P value** | | 0.166 | **0.016*** | | 0.083 | 0.516 | | | 0.738 | 0.382 | | **0.021*** |
|  |  | **Q value** | | 0.290 | 0.072 | | 0.193 | 0.602 | | | 0.738 | 0.535 | | 0.072 |
| **Medication duraiton** | | **R value** | | -0.329 | 0.021 | | -0.045 | 0.024 | | | 0.038 | -0.098 | | -0.030 |
|  |  | **P value** | | **0.013*** | 0.876 | | 0.739 | 0.860 | | | 0.779 | 0.469 | | 0.824 |
|  |  | **Q value** | | 0.088 | 0.876 | | 0.876 | 0.876 | | | 0.876 | 0.876 | | 0.876 |

*:P value or Q value <0.05.

DST, digit span test; LED, levodopa equivalent dose; MMSE, Mini-mental State; Examination; MoCA, Montreal Cognitive Assessment; SDMT, Symbol Digit Modality; SF, semantic fluency test.

**Supplementary Table 4. The significant cortical volume differences among four** **subgroups in treated patients**

| **Volume** | **G1: short duration with low LED**  **(N=20)** | **G2: long duration with low LED**  **(N=12)** | **G3: short duration with high LED**  **(N=10)** | **G4: long duration with high LED**  **(N=19)** | ***P value**** | **post-hoc (LSD) *P* value** | | | | | |
| --- | --- | --- | --- | --- | --- | --- | --- | --- | --- | --- | --- |
|  |  |  |  |  |  | **G1-G2** | **G1-G3** | **G1-G4** | **G2-G3** | **G2-G4** | **G3-G4** |
| Left SFC | 21498.65±2049.52 | 21348.08±1797.00 | 18855.90±2372.39 | 19655.00±1697.96 | 0.038 | 0.834 | **0.001** | **0.005** | **0.004** | **0.022** | 0.300 |
| Left rACC | 2557.15±371.74 | 2170.92±424.737 | 2268.20±306.69 | 2124.53±430.339 | 0.014 | **0.009** | 0.063 | **0.001** | 0.565 | 0.750 | 0.353 |
| *: controlling for age, gender, disease duration and TIV  Cortical volume is presented using mean±SD.  LED, levodopa dose equivalent dose; rACC, rostral anterior cingulate cortex; SFC, superior frontal cortex. | | | | | | | | | | | |

**Supplementary Table 5. The demographic and clinical data of follow-up patients at baseline and their last visit**

|  | **Baseline (N=23)** | **Last visit (N=23)** |
| --- | --- | --- |
| **Gender (M/F)** | 15 / 8 | |
| **Education (years)** | 8.52±3.65 | |
| **Age (years)** | 58.87±7.27 | 61.62±7.45 |
| **MMSE** | 28.00 (27.00-29.00) | 26.00 (24.00-28.00) |
| **MoCA^a^** | 23.00 (18.00-27.50) | 22.00 (20.00-26.00) |
| **Disease duration (years)** | 1.64 (0.67-2.92) | 3.96 (3.42-6.26) |
| **H-Y** | 2.50 (2.00-2.50) | 2.50 (2.00-3.00) |
| **UPDRS III** | 19.35±14.11 | 22.70±4.88 |
| **LED** | 0 | 400.00 (275.00-550.00) |
| **Medication duration** | 0 | 2.25 (1.25-4.01) |
| **TIV** | 1523881.576±137815.713414 | |
| a: results of 19 PD patients  F, female; H-Y, Hoehn–Yahr stage; LED, levodopa dose equivalent dose; M, male; MMSE, Mini-mental State Examination; MoCA, Montreal Cognitive Assessment; TIV, total intracranial volume; UPDRS, Unified Parkinson’s Disease Rating Scale. | | |

**Supplementary Table 6. Brain regions significantly correlated with LED in longitudinal data**

| **Region** | **LED** | |
| --- | --- | --- |
|  | **P value** | **Slope value (β)** |
| **Thickness** |  |  |
| lh_inferiortemporal | 0.021294 | -0.000148 |
| lh_lingual | 0.048970 | -0.000088 |
| lh_medialorbitofrontal | 0.047453 | 0.000112 |
| lh_middletemporal | 0.000066 | -0.000226 |
| lh_parsorbitalis | 0.001752 | -0.000125 |
| lh_parstriangularis | 0.018691 | -0.000092 |
| lh_rostralmiddlefrontal | 0.026367 | -0.000070 |
| lh_superiorfrontal | 0.006770 | -0.000101 |
| lh_superiortemporal | 0.005206 | -0.000145 |
| rh_isthmuscingulate | 0.017265 | -0.000191 |
| rh_superiortemporal | 0.008300 | -0.000095 |
| **Volume** |  |  |
| lh_lateralorbitofrontal | 0.004174 | -0.285455 |
| lh_middletemporal | 0.001574 | -0.786544 |
| **lh_superiorfrontal** | 0.001397 | -0.544461 |
| lh_superiortemporal | 0.004988 | -0.641962 |
| **rh_rostralmiddlefrontal** | 0.005183 | -0.409820 |

lh, left hemisphere; LED, levodopa dose equivalent dose; rh, right hemisphere.

Regions with bold type are brain regions with both significant inter-group differences in cross-sectional analyses and significant correlations with the LED in longitudinal analyses.

**Supplementary Table 7. Brain regions significantly correlated with medication duration in longitudinal data**

| **Region** | **Medcation duration** | |
| --- | --- | --- |
|  | **P value** | **Slope value (β)** |
| **Thickness** |  |  |
| lh_posteriorcingulate | 0.047861 | -0.048429 |
| rh_supramarginal | 0.039496 | -0.036317 |
| **Volume** |  |  |
| lh_parsorbitalis | 0.043565 | -74.227350 |
| lh_frontalpole | 0.008405 | -41.054795 |
| **rh_superiorfrontal** | 0.015582 | -409.679531 |

lh, left hemisphere; rh, right hemisphere.

Regions with bold type are brain regions with both significant inter-group differences in cross-sectional analyses and significant correlations with the medication duration in longitudinal analyses.


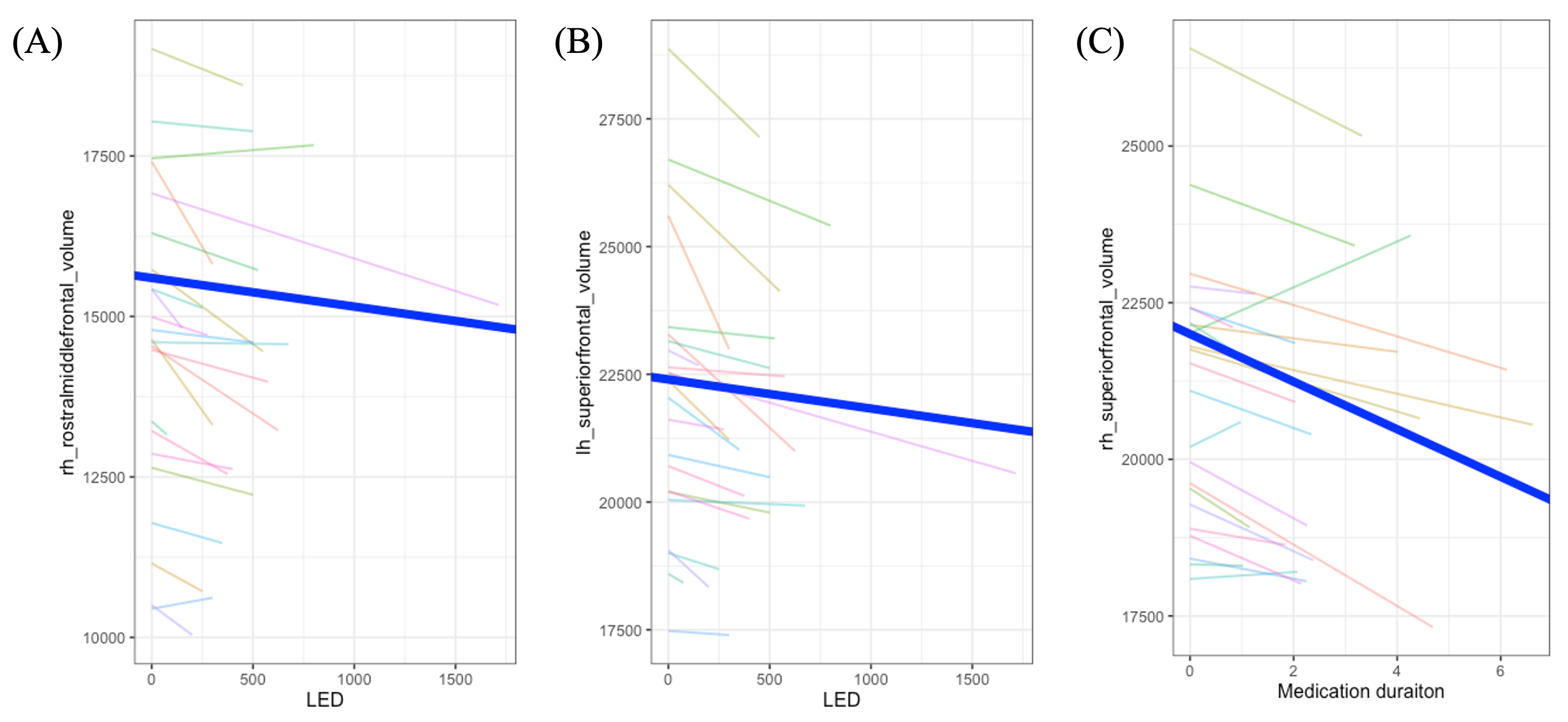


**Supplementary Figure 1. Longitudinal brain changes related to DRT in longitudinal data**

Longitudinal volume changes in the right rostral middle frontal cortex (A) and left superior frontal cortex (B) were significantly associated with LED. Longitudinal volume change in the right superior frontal cortex (C) was significantly associated with medication duration (in years).

Thick blue lines represent group-level changes, and the other lines represent individual changes.


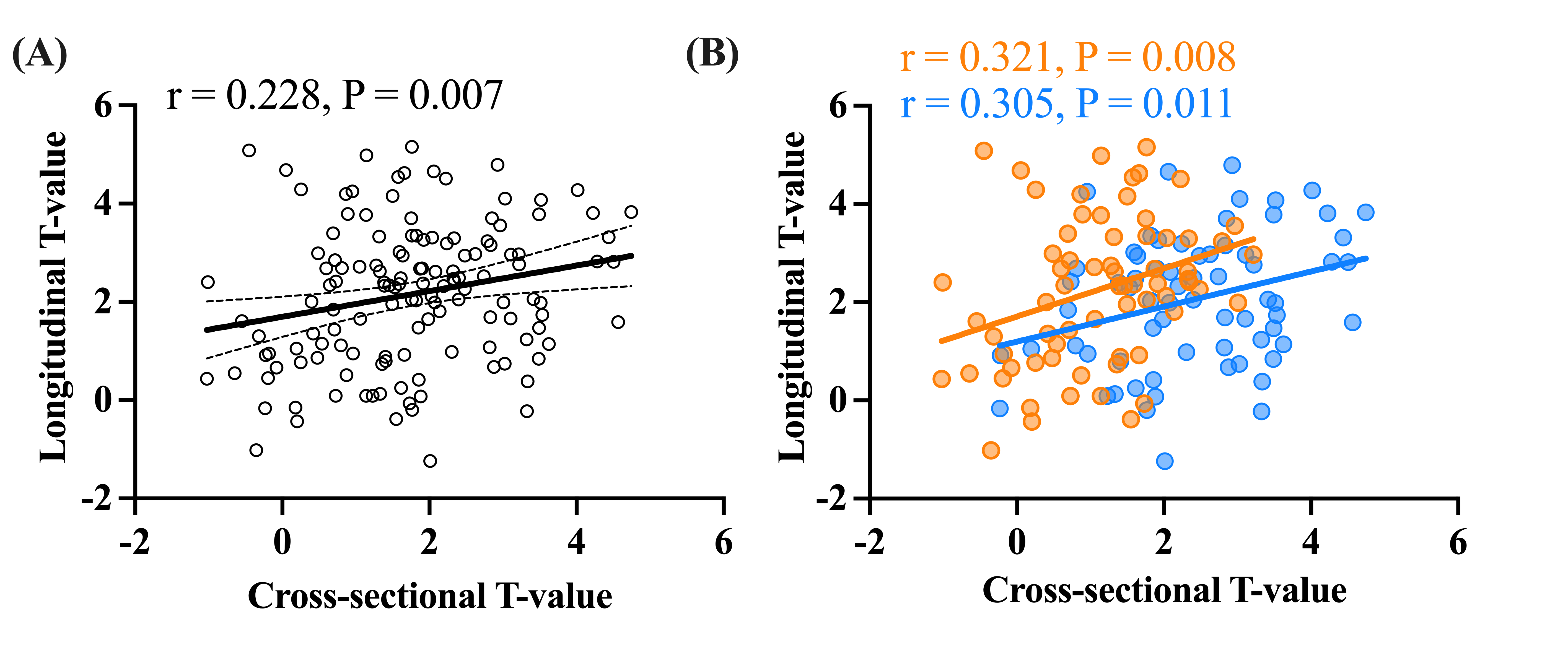


**Supplementary Figure 2. Correlation analyses between cross-sectional T-values and longitudinal T-values.**

(A) There was a significant correlation between cross-sectional T-values and longitudinal T-values in all 136 cortical structures. (B) The significant association persisted after conducting additional analyses on the 68 cortical structures relating to volume (in orange) and thickness (in blue), respectively.
